# Membrane Cholesterol in Skeletal Muscle: A Novel Player in Excitation-Contraction Coupling and Insulin Resistance

**DOI:** 10.1155/2017/3941898

**Published:** 2017-03-06

**Authors:** G. Barrientos, P. Sánchez-Aguilera, E. Jaimovich, C. Hidalgo, P. Llanos

**Affiliations:** ^1^Center for Molecular Studies of the Cell, Facultad de Medicina, Universidad de Chile, Santiago, Chile; ^2^Physiology and Biophysics Program, ICBM, Facultad de Medicina, Universidad de Chile, Santiago, Chile; ^3^Institute for Research in Dental Sciences, Facultad de Odontología, Universidad de Chile, Santiago, Chile; ^4^Cell and Molecular Biology Program, ICBM, Facultad de Medicina, Universidad de Chile, Santiago, Chile; ^5^BNI, Facultad de Medicina, Universidad de Chile, Santiago, Chile

## Abstract

Membrane cholesterol is critical for signaling processes in a variety of tissues. We will address here current evidence supporting an emerging role of cholesterol on excitation-contraction coupling and glucose transport in skeletal muscle. We have centered our review on the transverse tubule system, a complex network of narrow plasma membrane invaginations that propagate membrane depolarization into the fiber interior and allow nutrient delivery into the fibers. We will discuss current evidence showing that transverse tubule membranes have remarkably high cholesterol levels and we will address how modifications of cholesterol content influence excitation-contraction coupling. In addition, we will discuss how membrane cholesterol levels affect glucose transport by modulating the insertion into the membrane of the main insulin-sensitive glucose transporter GLUT4. Finally, we will address how the increased membrane cholesterol levels displayed by obese animals, which also present insulin resistance, affect these two particular skeletal muscle functions.

## 1. Introduction

The physiological relevance of plasma membrane cholesterol levels has attracted increased attention in recent years. Cholesterol is an essential component of eukaryotic membranes, which display molar ratios of cholesterol to phospholipids in the range of 7–55 mol% [1–4]. Physiological levels of cholesterol in the cellular membranes are critical to preserve membrane fluidity and thickness and to structure the lipid domains that are involved in signal transduction processes [2, 5].

Contraction of skeletal muscle takes place via the excitation-contraction (EC) coupling process [6]. Action potential propagation into the fiber interior through the transverse tubule (T-tubule) system initiates EC coupling, which results in the cytoplasmic Ca^2+^ increase that triggers muscle contraction [7]. In addition to contraction, insulin-sensitive glucose transport and glucose homeostasis represent additional key functions of skeletal muscle which occur predominantly at the level of the T-tubule system [8]. Accordingly, T-tubule composition and structure are likely to regulate both the EC process and insulin-sensitive glucose transport.

The mammalian T-tubule membranes are highly enriched in sphingomyelin and cholesterol compared to the surface sarcolemma [9]. This feature endows these membranes with a highly ordered lipid environment [10]. We have reported recently that single fibers isolated from adult skeletal muscle display a 26% decrease in cholesterol content following incubation with the cholesterol removing agent methyl-*β*-cyclodextrin (M*β*CD). This agent also alters the distribution pattern of the voltage-dependent calcium channel Cav1.1 in T-tubules and suppresses electrically evoked Ca^2+^ transients [11].

Skeletal muscle is the largest body organ in nonobese subjects and represents the major site of insulin-stimulated glucose disposal [12]. Insulin increases glucose uptake into skeletal muscle and adipose tissue by redistributing type 4 glucose transporters (GLUT4) from their intracellular location to the plasma membrane [13]. In skeletal muscle, insulin resistance (IR) is associated with disturbed insulin signaling, leading to defective GLUT4 traffic to the T-tubules and the surface membrane [8].

Animals fed a high-fat diet (HFD) become insulin-resistant and exhibit elevated levels of membrane cholesterol compared with normal chow-fed animals [14]. Likewise, mice fed a HFD become obese, develop IR, and display increased levels of cholesterol in isolated skeletal muscle T-tubule membranes and adult muscle fibers [15]. Conversely, new insights into GLUT4 trafficking reveal that compounds that partially reduce membrane cholesterol content increase insulin-independent GLUT4 translocation and glucose uptake, both in adipocytes [16] and in muscle cell lines [14]. Therefore, altering the physiological levels of membrane cholesterol may lead to cellular malfunction and thus may contribute to the pathological processes triggered in humans by obesity or by cholesterol depletion caused by pharmacological agents. Here, we will review the critical evidence that supports a role of membrane cholesterol as a new player in physiological muscle function and in the IR condition.

## 2. Role of Cholesterol in Plasma Membrane Properties

Cholesterol is the single most abundant lipid molecule of plasma membranes, representing up to 55 mol% of the total lipid composition [1–4]. The cholesterol molecule is essential for membrane biogenesis [4] and influences the structure and physical properties of biological membranes, including membrane thickness [17] and fluidity [18]. Membrane cholesterol participates in a wide range of physiological functions including limiting ion leakage through membranes [19], modulation of signal transduction pathways [5], and traffic of membrane proteins [20].

**Figure 1:**
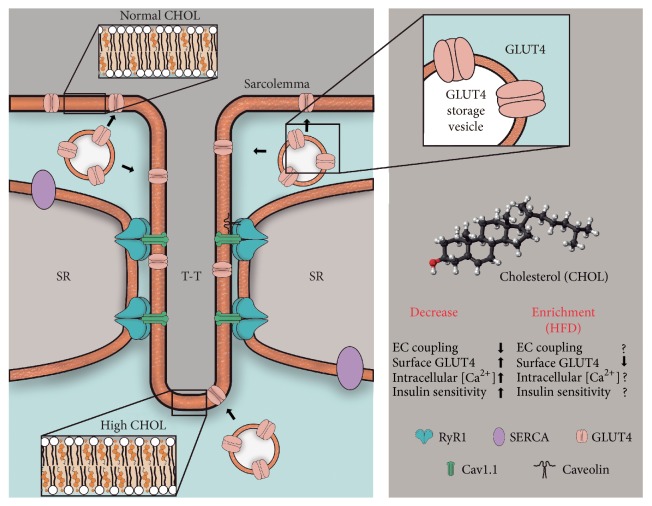
Schematic figure showing the T-tubule (T-T) system and its high cholesterol content compared with the surface membrane (sarcolemma, see inserts). Note the main proteins of the EC coupling complex and the sites of GLUT4 translocation. In response to insulin, the GLUT4 transporters translocate mainly to the T-tubule system, where EC coupling takes place, and also to the surface membrane region. Modifications of cholesterol content affect both EC coupling and GLUT4 mediated glucose transport in skeletal muscle. ↑: increment; ↓: decrease; ?: unknown effect.

Cholesterol is a polycyclic amphipathic molecule derived from a sterane backbone (Figure 1). It has a polar head formed by a single hydroxyl group which in membranes can form hydrogen bonds with polar groups of proteins or lipids [21]. The nonpolar section of cholesterol has two faces, a planar face called the *α*-surface and a rough face called the *β*-surface. These sections allow specific cholesterol interactions with protein *α*-helical regions and *β*-surfaces [21, 22].

Cholesterol modifies the organization of lipids in artificial bilayers. Phosphatidylcholine molecules with unsaturated hydrocarbon chains can adopt a liquid-disordered fluid phase in bilayers; however, cholesterol addition induces a change to the liquid-ordered phase, decreasing the fluidity of the membrane [23–25]. The spatial distribution of cholesterol responds according to the umbrella model, whereby the polar head groups of phospholipid function as an umbrella, shielding the hydrophobic moiety of cholesterol molecules from water [26]. Cholesterol has higher affinity for sphingolipids, leading to a highly regular distribution in membranes which minimizes cholesterol-cholesterol contact [26].

Lipid rafts are small-scale (10–200 nm) domains found in live cell membranes enriched in cholesterol and sphingolipids. In lipids rafts, the interaction of cholesterol with sphingolipids is more stable; cholesterol presents its *α*-surface to these lipids leaving its *β*-surface to interact with transmembrane domains of integral proteins [21]. Lipid rafts play an essential role in membrane-protein sorting and in the formation of signaling complexes [27–29]. In these lipids domains, cholesterol increases the order of lipid-acyl chains, increasing the local membrane thickness and limiting the type of integral membrane proteins located on this hydrophobic environment. These changes around membrane proteins can modulate the local lipid environment and modify the internal protein conformation states and their function [30].

A previous report [31] described a Cholesterol Recognition/Interaction Amino Acid Consensus sequence (CRAC domain), which is a short linear amino acidic motif with a specific vectorial direction (from N- to C-terminal). The CRAC domain starts at the N-terminus with a Leu (L) or Val (V) residue, followed by a segment comprising 1 to 5 residues. The segment continues with a mandatory Tyr (Y) residue, a segment comprising 1 to 5 residues, and ends with a basic Lys (K) or Arg (R) residue, (L/V)-X_1–5_-(Y)-X_1–5_-(K/R) [32]. CRAC domains interact with cholesterol in the cytoplasmic leaflet of the membrane. These motifs belong to some transmembrane protein domains and have a favorable fit for cholesterol binding [21].

Another newly recognized cholesterol-binding sequence, known as CARC domain, has recently been described; it has almost the same sequence as CRAC but runs in the opposite direction (from C- to N-terminal) and has a central aromatic amino acid, which can be either Tyr or Phe, (K/R)-X_1–5_-(Y/F)-X_1–5_-(L/V) [33]. In both cases, the van der Waals forces and H-bonds between the Y residue and the OH group of the cholesterol molecule participate in the interaction between cholesterol and the CRAC/CARC-containing protein [21].

Both CRAC and CARC motifs represent oriented amino acid sequences, with an apolar amino acid residue at one terminal and a highly polar, positively charged basic residue at the other end [32]. In some cases, these amino acidic sequences are located in the same transmembrane segment but CARC is associated with the outer leaflet and CRAC is located at the inner leaflet of the plasma membrane. There are several examples of membrane receptors that have such a dual interaction with CRAC and CARC. These receptors include neuropeptide FF receptor, metabotropic glutamate receptor 5, GABA type B receptor subunit 2, CB1 receptor, 5-HT7 receptor, adenosine receptor A1, VIP receptor 1, prolactin-releasing peptide receptor, oxytocin receptor, TRVP1 receptor, and corticotrophin-releasing factor receptor 1 [34].

The alignment of the dipolar components of phospholipids at the water interface generates a membrane-internal potential known as the dipole potential [35], with an estimated magnitude around 280 mV [36]. It has been suggested that the dipole potential modulates the translocation rates of ions across lipid membranes [37]. The activities of Na^+^/K^+^-ATPase and phospholipase A2 increase with increasing dipole potential [38, 39]. Cholesterol intercalation strongly affects the dipole potential; cholesterol removal generates a reduction close to 50 mV [40]. Restoration of cholesterol levels reverses this reduction in natural membranes [41]. However, cholesterol levels have negligible effects on the dipole potential exhibited by polyunsaturated membranes [42]. Currently, there is no information to our knowledge regarding the effects of obesity, metabolic syndrome, or cholesterol lowering treatments on dipole potential.

The manipulation of membrane cholesterol content is useful to study its effects on cellular physiology. The most common and simple approach has been to treat membranes with cyclodextrins, a family of cyclic compounds, which have a central hydrophobic pocket that extracts cholesterol from the cell membranes [43]. To date, however, only few studies, some of which are presented below, have provided information on how cholesterol modulation affects skeletal muscle protein functionality.

## 3. T-Tubule Structure and Composition

The T-tubule system of skeletal muscle is an intricate network composed of narrow tubules of around 40–85 nm in diameter which originate from deep invaginations of the surface plasma membrane [44]. This membrane system represents around 80% of the total plasma membrane surface of skeletal muscle [45]. Early studies using differential centrifugation reported that mammalian T-tubule membranes have a high proportion of cholesterol and sphingolipids [9] which resemble the composition of cholesterol-enriched lipid rafts domains [11]. Moreover, electron paramagnetic resonance assays indicate that at physiological temperature the lipid phase of T-tubule membranes is remarkably less fluid than that of other mammalian plasma membranes [10], resembling the low fluidity of thermophilic bacterial membranes. The T-tubules contain many proteins involved in EC coupling and other signaling processes; membrane cholesterol levels modulate the function of several of these proteins, including Cav1.1 [46], caveolin-3 [47], and Na^+^/K^+^- ATPase [48].

## 4. T-Tubule Cholesterol Levels Influence the EC Coupling Process

During muscle contraction, the action potential elicited at the neuromuscular junction propagates through the surface membrane into the T-tubule network, which is a key element in the EC coupling process [7]. The T-tubule membrane is flanked by two junctional sarcoplasmic reticulum (SR) membranes, forming structures known as triads which allow the direct interaction of the T-tubule residing protein Cav1.1 with Ryanodine receptor type 1 (RyR1), an integral SR membrane protein [7]. During EC coupling, Cav1.1 works as voltage sensor and commands transient RyR1 opening in response to membrane depolarization; the subsequent Ca^2+^ release from the SR produces muscle contraction [6].

It has been proposed that membrane cholesterol can modulate Cav1.1 activity [46]. In mechanically skinned fibers, in which the surface membrane is removed leaving the T-system intact, cholesterol depletion with M*β*CD induces T-tubule system depolarization without changes in its integrity [49]. Moreover, cholesterol depletion from intact fetal skeletal muscle using M*β*CD decreases Cav1.1 Ca^2+^ currents and shifts their voltage dependence to more positive values; it is important to remark that M*β*CD saturated with cholesterol does not affect Cav1.1 function [46].

We have shown that partial cholesterol removal from dissociated adult fibers inhibits EC coupling and depolarizes the fibers [11]. Cholesterol removal with M*β*CD also increases the resting Ca^2+^ level, apparently by stimulating Ca^2+^ release from internal stores [11], suggesting that plasma membrane cholesterol might modulate functional interactions of the T-tubule membranes with the intracellular Ca^2+^ stores.

Caveolin-3 is a cholesterol-binding protein [47], which directly interacts with Cav1.1 [50] and modulates its Ca^2+^ channel function [51]. A recent report showed that cholesterol also modulates cardiac EC coupling and contraction [52]. Collectively, these results raise the possibility that cholesterol modulates striated muscle EC coupling by direct interaction with the protein complex engaged in this process and indirectly by modulating the lipid environment and accessory proteins such as caveolin-3.

## 5. Cholesterol: A Novel Regulator in GLUT4 Translocation

Skeletal muscle is a major contributor to whole-body metabolism; it is the largest insulin-sensitive tissue in the body, which makes it a key locus for insulin-stimulated glucose uptake [53]. In humans under euglycemic, hyperinsulinemic conditions, around 80% of body glucose uptake occurs in skeletal muscle, which represents a central component of glucose homeostasis [12]. In addition, this tissue is also an important consumer of fatty acids, which together with glucose constitute the principal energy sources of skeletal muscle [54].

The glucose transporter GLUT4 is one of fourteen members of the glucose transport family and displays high affinity for glucose [55]. Patients with IR and type 2 diabetes mellitus (T2DM) show defects in insulin-stimulated glucose metabolism in skeletal muscle. These alterations have been attributed to a disturbance in glucose transport, resulting mainly from dysregulated GLUT4 trafficking, the predominant insulin-sensitive glucose transporter expressed in skeletal muscle [56–58]. Both insulin and muscle contraction induce GLUT4 translocation to the skeletal muscle plasma membrane [59], presumably by engaging separate signaling pathways. The increase of surface GLUT4 occurs as a result of translocation of GLUT4-containing intracellular vesicles to the plasma membrane [60].

In order to enter into the muscle cell, glucose delivered by blood flow must be transported across the surface membrane and the T-tubule membranes into the cytoplasm, where it is trapped by hexokinase II action [58]. Insulin binding to the insulin receptor (InsR) promotes a conformational change in the receptor which leads to the transphosphorylation in tyrosine residues of its cytoplasmic *β* subunits [61]. The activated InsR phosphorylates, among other proteins, the main InsR substrate (IRS) proteins, including IRS-1 and IRS-2. Tyrosine-phosphorylated IRS-1 and IRS-2 serve as docking sites for SH2 domain-containing proteins, such as class IA (p85/p110-type) phosphatidylinositol 3 kinase (PI3K) [62]. The activation of this lipid kinase increases phosphatidylinositol-3,4,5-trisphosphate (PIP3) levels at the inner face of the plasma membrane and recruits pleckstrin (PH) homology domain-containing proteins, which are essential for insulin-stimulated GLUT4 translocation and the ensuing glucose uptake [8]. The PH domain-containing Akt protein is a cytoplasmic serine-threonine kinase that plays a fundamental role in mediating insulin-stimulated GLUT4 translocation; Akt organizes several downstream molecules that involve successive steps, including AS-160 activation and Rab Family GTPases, which finally position GLUT4 in the plasma membrane and promote GLUT4-mediated glucose transport [13, 63]. The increase in plasma membrane GLUT4 occurs due to a large increase in the rate of GLUT4 exocytosis, coupled with a smaller decrease in the rate of GLUT4 endocytosis [60]. The continuous recycling of GLUT4 offers the flexibility to regulate both its exocytic and endocytic rates. In cultured adipose and muscle cells, insulin rapidly stimulates the rate of exocytosis of GLUT4 transporters [64]. However, there is limited information about the mechanisms that regulate GLUT4 endocytosis. Although insulin reduces GLUT4 endocytosis in adipose cells [65], it does not affect the rate of GLUT4 internalization in rat cardiomyocytes [66] or in skeletal muscle cell lines [67]. In adult skeletal muscle, GLUT4 accumulates in several intracellular compartments, and although it locates preferentially in perinuclear regions, it is also present in peripheral vesicles [68, 69]. Functional studies in adipocytes and skeletal muscle cell lines indicate that insulin-derived cellular signals promote GLUT4 translocation to the plasma membrane from a specialized compartment termed GLUT4 storage vesicles [60].

Various proteins including actin, actin dynamics, and microtubular motors intricately regulate the process of GLUT4 vesicle mobilization, tethering, docking, and fusion in response to insulin. GLUT4 is internalized via clathrin-mediated endocytosis or via cholesterol-dependent but clathrin-independent endocytosis [60]. However, there are few studies addressing the role of membrane cholesterol in GLUT4 traffic.

In adipocyte and skeletal muscle cell under basal conditions, around 5% of GLUT4 is present in the surface membranes; insulin stimulation increases its level to about 50% [70]. Early studies by nuclear magnetic resonance [71, 72] complemented with more recent reports have shown that GLUT4 translocation is defective in T2DM patients [8, 73]. The majority of GLUT4-containing vesicles do not move long distances but are depleted locally in the surface membrane or T-tubule regions [8, 74]. Moreover, analysis of GLUT4 translocation in insulin-resistant muscle showed that GLUT4 recruitment is affected primarily in the T-tubule region [8]. Muscles subjected to osmotic shock to dissociate the T-tubule connection with the surface membrane have their T-tubule network with no access to insulin and glucose from the extracellular fluid [8, 75]. The dissociation of the T-tubule system reduces basal glucose transport by 50% and completely abolishes the insulin-induced increase in glucose transport [8], highlighting the critical role of the T-tubule system in insulin-mediated glucose transport [75]. Nevertheless, it is not clear why the dissociation of the T-tubule system inhibits insulin-dependent glucose transport through the surface membrane.

The mammalian T-tubule membranes are highly enriched in cholesterol and sphingolipids [9] endowing them with a rigid lipid environment with highly restricted membrane fluidity properties [10]. GLUT4 translocation occurs at cholesterol-rich microdomains [76], suggesting that changes in cholesterol levels modulate insulin-stimulated GLUT4 exocytosis. In fact, current evidence supports the hypothesis that increased plasma membrane cholesterol levels have a key role in the impaired GLUT4 traffic observed in IR and T2DM, since glucose-intolerant animal models and humans accumulate cholesterol in their skeletal muscle membranes [14]. Recently, using HFD-fed animals as a model of IR, we reported that triad-enriched fractions isolated from the skeletal muscle of these obese animals have around 30% higher cholesterol content than triads from lean control animals [15]. In addition, muscle fibers isolated from HFD-fed obese mice show a 40% decrease in insulin-stimulated glucose uptake rates compared to fibers from lean control mice. In HFD-fed mice, four subcutaneous injections of M*β*CD improved their defective glucose tolerance test, normalized their high fasting glucose levels, and restored insulin-stimulated glucose uptake in adult skeletal muscle fibers [15]. In addition, preincubation of isolated muscle fibers with relatively low concentrations of M*β*CD increases both basal glucose uptake and insulin-induced glucose uptake in fibers from controls or HFD-fed mice. In muscle fibers from HFD-fed mice, M*β*CD improves insulin sensitivity and Indinavir, a GLUT4 antagonist, prevents the stimulatory effects of M*β*CD on glucose uptake [15]. In addition, M*β*CD increases membrane GLUT4 content and elicits intracellular calcium signals that are inhibited by Dantrolene, an agent which blocks the functional interaction of Cav1.1 and RyR1 [77] and reduces M*β*CD-mediated glucose uptake [15]. Interestingly, L6 myotubes cultured in a hyperinsulinemic medium resembling in vivo conditions that promote the progression of insulin resistance display an increase in membrane cholesterol [78]. The increased cholesterol levels in the plasma membrane of L6 myotubes cells may impair insulin action through a loss of cortical filamentous actin (F-actin), leading to defective GLUT4 regulation by insulin and an increase in the hexosamine biosynthesis pathway [14].

Treatment with chromium picolinate, a compound that removes membrane cholesterol, activates GLUT4 trafficking and enhances insulin-stimulated glucose transport via a cholesterol-dependent mechanism [79]. In addition, chromium supplementation significantly improves fasting glycemia in T2DM patients [80]. Treatment with M*β*CD reversibly decreases the cholesterol content of membranes in a dose-dependent manner, leading to increased GLUT4 incorporation into the plasma membrane of L6 myotubes [14]. More recently, it has been reported that AMP-activated protein kinase (AMPK) enhances insulin-stimulated GLUT4 regulation via lowering membrane cholesterol levels [78]. All together, these reports suggest a novel aspect of GLUT4 regulation by cholesterol in skeletal muscle.

## 6. Concluding Remarks and Perspectives

The cholesterol content of the T-tubule membrane in skeletal muscle is significantly higher than the levels present in the plasma membrane of most cells. A decrease in membrane cholesterol with cholesterol-removing agents alters muscle function, affecting both the excitation-contraction coupling process and glucose transport mediated by GLUT4 translocation to the T-tubule membrane (Figure 1). In conditions such as high-fat diet-induced obesity, the cholesterol content of T-tubule membranes increases even further, making it likely that pathological conditions, such as insulin resistance and type 2 diabetes, entail increased T-tubule cholesterol content. Accordingly, restoring membrane cholesterol levels in the T-tubule system via increasing surface GLUT4 levels in response to insulin may constitute an interesting therapeutic target to ameliorate insulin resistance.
